# Exploring the Antioxidant and Structural Properties of Black Bean Protein Hydrolysate and Its Peptide Fractions

**DOI:** 10.3389/fnut.2022.884537

**Published:** 2022-06-06

**Authors:** Yin Chen, Zhaojun Zheng, Zixuan Ai, Yan Zhang, Chin Ping Tan, Yuanfa Liu

**Affiliations:** ^1^State Key Laboratory of Food Science and Technology, School of Food Science and Technology, National Engineering Research Center for Functional Food, National Engineering Laboratory for Cereal Fermentation Technology, Collaborative Innovation Center of Food Safety and Quality Control in Jiangsu Province, Jiangnan University, Wuxi, China; ^2^Department of Food Technology, Faculty of Food Science and Technology, Universiti Putra Malaysia, Seri Kembanganr, Malaysia; ^3^Future Food (Nanjing Fuzhe) Research Institute Co., Ltd., Nanjing, China

**Keywords:** antioxidant activities, black bean protein, enzymatic hydrolysis, peptide fractions, response surface methodology

## Abstract

A great deal of attention has been paid to charactering the protein hydrolysates prepared by enzymatic hydrolysis, while the influence of molecular weight (MW) distributions on the resultant hydrolysates remains unclear. This study aimed to explore the physicochemical and antioxidant characteristics of protein hydrolysate and its peptide fractions. Bromelain has been commonly used to hydrolyze black bean protein *via* response surface methodology (RSM). The optimal hydrolysis parameters were observed at 52°C, pH 7, E/S ratio of 2.2 (ratio of enzyme to substrate), and 4 h. Under these parameters, the hydrolysate (BPH) presented DPPH radical scavenging activity and Fe^2+^ chelating activity with IC_50_ values of 100.08 ± 2.42 and 71.49 ± 0.81 μg/mL, respectively. This might be attributed to structural characteristics, varying with different molecular weight distributions. Interestingly, among BPH and its peptide fractions, peptides smaller than 3 kDa were noted to exhibit the strongest DPPH and ABTS radical scavenging activity. More intriguingly, this peptide fraction (<3 kDa) could predominantly prolong the induction period of sunflower oil, which was, respectively increased to 1.31 folds. This may be due to high proportions of hydrophobic amino acids. Unexpectedly, the optimal Fe^2+^ chelating activity was observed in the peptide fraction measuring at 3–10 kDa, showing highly positive correlations with histidine and arginine. These identified peptide fractions derived from black bean protein can therefore be employed for food fortification acting as natural antioxidant alternatives.

## Introduction

The consumption pattern of plant-based food has seen a rapid growth due to pursuits of healthy living, environmental awareness, and animal welfare (1). As an irreplaceable food ingredient, plant protein has attracted a great deal of attention recently. Black bean (*Phaseolus vulgaris L.*) has been recognized as an optimal source of high-quality protein (32–43.6%), potentially exerting numerous biological benefits (2). Under different enzymatic hydrolysis process conditions, the black soybean peptide prepared from black soybean protein can effectively improve the physical and chemical properties of the original protein, or be endowed with various biological activities such as anti-oxidation, the reduction of blood glucose, anti-cancer, and lowering blood lipids. However, the bioactive peptides are generally enclosed inside the black bean protein. Therefore, it is essential to find an approach for exploring the biological potential of peptide fractions from the black bean protein.

Enzymatic hydrolysis is a highly efficient and environment-friendly approach to cleave plant proteins to release biologically active peptide fractions. Four proteolytic enzymes hydrolyzed brewer grain yeast proteins to release protein hydrolysates, which exhibited excellent DPPH scavenging and ferric-reducing activities (3). Likewise, trypsin-treated protein hydrolysates from the palm kernel cake were found to strongly scavenge DPPH and ABTS radicals (4). Accordingly, enzymatic hydrolysis endows the plant-based protein hydrolysates with antioxidant properties, which are deemed beneficial to forestall lipid peroxidation as well as to enhance dietary nourishment (5). Given the crucial influence of hydrolysis factors (temperature, pH, time, and E/S, etc.), on enzymatic hydrolysis, process optimization has been identified to be a wise alternative for achieving the desirable antioxidant effects of hydrolysates. Response surface methodology (RSM) is well-documented as an effective and practical tool for optimizing hydrolysis conditions.

Hydrolysis plays a crucial role in the antioxidant function of protein hydrolysates by changing structural characteristics like molecular weight (MW). For instance, rice bran protein hydrolysates were fractionated into three peptide fractions (≤5, 5–10, and ≥10 kDa), of which the ≤5 kDa fraction exhibited the highest antioxidant activity (6). According to Zou et al. (7), peptide fractions with MW distributions below 1 kDa demonstrated the highest oxygen radical absorbance capacity among the four fractions derived from wheat bran protein hydrolysates. Consequently, the antioxidant activities of plant-based protein hydrolysates have been closely associated with their MW distributions. However, the relationship between MW distribution and antioxidant activity of protein hydrolysate remains undefined. In particular, each protein hydrolysate is known to possess a special MW distribution, further emphasizing the importance of exploring antioxidant peptide fractions.

Although black bean has been considered as an excellent protein resource for preparing hydrolysates, the specific antioxidant peptide fractions are yet to be characterized. In this study, we employed bromelain to hydrolyze black bean protein. We also optimized the hydrolysis conditions for obtaining the protein hydrolysate with optimal antioxidant capacity. To further exploit the relationship between activity and MW, the optimal hydrolysate was fractionated into three peptide fractions. These fractions were investigated for their antioxidant activities as well as structural properties to reveal the correlation of activity with MW distribution.

## Materials and Methods

### Materials

Black bean protein was provided by Shiyue Daotian company (Beijing, China). Bromelain was purchased from J&K Scientific Co., Ltd. (Shanghai, China). 2,2-Diphenyl-1-picrylhydrazyl (DPPH), 2,2′-azino-bis, 3-ethylbenzothiazoline-6-sulfonic acid (ABTS), ferrozine, and 1-anilinonaphthalene-8-sulfonic acid (ANS) were purchased from Sigma-Aldrich. All other chemicals and reagents were of analytical grade.

### Extraction of Black Bean Protein

Black bean protein was prepared according to the method published by Zheng et al. (8), with slight modification. First, black beans were ground into powder using a high-speed grinding machine (Tianjin Instrument Co., Ltd., Tianjin, China). The black bean powder was then defatted twice using hexane (1:3, w/v), for 3 h each time. After air-drying overnight, the solid powder was added to deionized water at a ratio of 1:10 (w/v). The pH was then adjusted to 8.0 using 1 M NaOH. After stirring for 2 h at room temperature, the mixture was centrifuged at 3,000 × *g* for 20 min to obtain the supernatant. The pH value of the solution was adjusted to 4.5 using 1 M HCl. After centrifugation at 3,000 × *g* (4°C) for 15 min, the precipitate was re-dissolved into deionized water and adjusted to a neutral pH. Finally, the protein powder was freeze-dried. The preparation of the control group resembled that of the protein hydrolysate, without the addition of the inactivated protease.

### Optimization of Enzymatic Hydrolysis

To prepare the hydrolysates with the most effective antioxidant activity, RSM was used to optimize the hydrolysis parameters of black bean by bromelain. A central composite design was created and conducted to optimize enzymatic hydrolysis. Temperature, pH, E/S, and time were identified as independent variables, and their ranges were chosen based on preliminary experiments and manufacturer information. Specifically, the coded values were −1, 0, and 1, for hydrolysis temperatures of 50, 55, and 60°C (*x_1_*), respectively. The pH values were 6.5, 7, and 7.5 (*x_2_*). The enzyme concentrations were 1.5, 2, and 2.5% (*x_3_*). The hydrolysis times were 3, 4, and 5 h (*x_4_*). The proposed response surface model can be illustrated by the following equation:


(1) $$y=b_{0}+\sum_{i=1}^{4}b_{i}\,x_{i}+\sum_{i=1}^{4}b_{i\,i}\,x_{i}^{2}+\sum_{i=1}^{3}\sum_{j=i+1}^{4}b_{i\,j}\,x_{i}\,x_{j}$$

### Preparation of Peptide Fractions With Different Molecular Weights

The black bean protein hydrolysate was fractionated into three fractions using 3 and 10 kD ultrafiltration membranes (Millipore Corporation Co., Ltd, Billerica, MA, United States). Three different fractions with molecular weight distributions of >10, 3–10, and <3 kDa were labeled as BPH (>10 kDa), BPH (3–10 kDa), and BPH (<3 kDa), respectively.

### DPPH Radical Scavenging Activity

All samples were dissolved in deionized water to a final concentration of 2 mg/mL. 1 mL of sample and 400 μL of DPPH solution were reacted at room temperature. Following this, the solutions were scanned at wavelengths from 475 to 800 nm using a UV-visible spectrophotometer (Ultrospec 7000, GE Healthcare, Chicago, IL United States).

DPPH radical scavenging activity was determined according to the method described by Zheng et al. (8). Briefly, 50 μL BPH (15.62–500 μg/ml) and 50 μL DPPH solution (0.1 mM in ethanol) were added to each well of a 96-well plate. Additionally, 50 μL enzymatic hydrolysates, 50 μL 95% ethanol (the control group), 50 μL DPPH, and an equivalent volume of deionized water (the blank group) were added. Each sample was duplicated. After 30 min at room temperature, the absorbance was measured at 517 nm using a microplate reader (SpectraMax 190, Molecular Devices, San Jose, CA, United States). DPPH radical scavenging activity was then calculated using the following formula:


(2) $$DPPHradicalscaveningactivity\left(\%\right)=\left(\frac{1-\left(A_{1}-A_{2}\right)}{A_{3}}\right)\times 100$$

where *A_1_* is the absorbance of the sample, *A_2_x* is the absorbance of the control, and *A_3_* is the absorbance of blank.

### ABTS Radical Scavenging Activity

All samples were dissolved in deionized water to a final concentration of 0.5 mg/mL. 1 mL of each sample and 300 μL of ABTS solution were mixed and measured in the wavelengths range of 475–800 nm, using a UV-visible spectrophotometer (Ultrospec 7000, GE Healthcare, Chicago, IL, United States).

ABTS radical scavenging activity was performed as described by Ngoh and Gan (9), with slight modification. The solution of ABTS radicals was prepared with 7 mM ABTS and 2.45 mM potassium per-sulfate and stored in the dark for 16 h at room temperature. This solution was diluted to an absorbance of about 0.8 (734 nm) to prepare the ABTS working solution. 50 μL of each sample was mixed with 150 μL of ABTS working solution, and then read at 734 nm after 6 min. Meanwhile, 50 μL of sample with 150 μL of deionized water was defined as the control group. 50 μL of deionized water with 150 μL of ABTS solution was defined as the blank group. The ABTS inhibition (%) was calculated using the following formula:


(3) $$ABTSinhibtion\left(\%\right)=\left(\frac{1-\left(A_{1}-A_{2}\right)}{A_{3}}\right)\times 100$$

### Metal Chelating Activity

Peptides exert antioxidant effects mainly by scavenging free radicals and chelating metal ions. Therefore, chelating metal ions is also one of the main methods for assessing the antioxidant capacity of peptides.

Each sample was diluted to a final concentration of 1 mg/mL. Meanwhile, 300 μL of solution was mixed with 500 μL of FeCl_2_ (200 μM) and 500 μL of 5 mM ferrozine at room temperature. The reaction solution was scanned at wavelengths ranging from 475 to 800 nm using a UV-visible spectrophotometer (Ultrospec 7000, GE Healthcare, Chicago, IL, United States).

The ability to chelate the ferrous ion (Fe^2+^) was measured using a slightly modified version of the method reported by Zheng et al. (8). Briefly, 50 μL of sample was added into a 96-well plate with both 100 μL of FeCl_2_ solution (20 μM) and 100 μL of 0.5 mM ferrozine. Then, 50 μL of deionized water without any sample was used as the control group, while 200 μL of deionized water was used as the blank group instead of the reaction solution. The absorbance was measured at 562 nm after incubation for 10 min. The Fe^2+^ chelating activity was calculated using the following formula:


(4) $${\text{Fe}}^{2+}\mathrm{chelating}\mathrm{activity}\left(\%\right)=\left(\frac{1-\left(A_{1}-A_{2}\right)}{A_{3}}\right)\times 100$$

### Surface Hydrophobicity

1-Anilinonaphthalene-8-sulfonic acid (ANS) fluorescence was used to measure surface hydrophobicity. Samples were analyzed at concentrations ranging from 0.005 to 0.20 mg/mL with 0.01 M phosphate buffer (pH 7.0). 50 μL of 8 mM ANS solution was added to 4 mL of each sample dilution. The fluorescence intensity (FI) of the sample solution was measured using an F-7000 fluorescence spectrophotometer (Hitachi, Japan) at excitation and emission wavelengths of 365 nm and 484 nm, respectively. Surface hydrophobicity was expressed as the initial slope (H_0_) of the FI versus concentration (%) plot.

### Ultraviolet-Visible Spectra

Ultraviolet-Visible (UV) absorption of the hydrolysate was measured according to a method shared by Huang et al. (10), with slight modifications. Samples were dissolved in deionized water to a final concentration of 0.1 mg/mL. All samples were subjected to absorbance at wavelengths between 200 and 800 nm using a UV-visible spectrophotometer (Ultrospec 7000, GE Healthcare, Chicago, IL, United States).

### Circular Dichroism

The measurement of Circular Dichroism (CD) spectra was conducted according to the description by Mohammed et al. (11) using a Chirascan CD Spectrometer (Applied Photophysics Ltd., Leatherhead, United Kingdom). Briefly, protein hydrolysates were diluted in deionized water to a final concentration of 0.10 mg/mL. Each sample was added to a 1 mm quartz cuvette at room temperature. Samples were then scanned at wavelengths ranging from 190 to 260 nm with a scan speed of 100 nm/min and an interval of 0.5 nm.

### Fluorescence Spectroscopy

A fluorescence spectrophotometer (F-7000, Hitachi, Tokyo, Japan) was used to measure the fluorescence spectrum of protein hydrolysates. Briefly, 1.5 mL of protein hydrolysates (2 μg/mL) was prepared in 10 mM phosphate buffer (pH 7.0) and mixed with 20 μL of ANS stock solution (8 mM). The excitation wavelength was set to 390 nm, and the emission wavelength was set to 470 nm. The mixture was generated by scanning from 400 to 600 nm at an emission interval of 5 nm.

### Antioxidant Activity of Hydrolysates in Sunflower Oil

Antioxidant activity of black bean hydrolysates on the oxidation stability of sunflower oil was detected by means of rancimat test, using an 892 Professional Rancimat Instrument (Metrohm Ltd., Herisau, Switzerland). Sunflower oil (3 g) was mixed with 0.5% sample (the protein hydrolysates were stored at −80°C and lyophilized) in glass test tube. Then the induction time of sunflower oil with or without the addition of hydrolysate was detected under constant airflow of 20 L/h within the thermostat-controlled block heater at 120 ± 1.6°C. The experiments were carried out at least in duplicate.

### Amino Acid Profiles

The amino acid content in samples was analyzed by HPLC coupled with ODS amino acid column (4.6 mm × 250 mm, Agilent 1100, Agilent Technologies, Santa Clara, CA, United States). First, 4 mL of hydrolysate was precipitated by adding an equivalent volume of 6 M HCl to the sample. After incubation for 22 h at 4°C, the sample was filtered through a 0.22 mm filter membrane and was thereafter centrifuged for 10 min at 4,000 × *g*. Finally, the solution was collected, and the amino acid composition was determined by HPLC.

SIMCA-P 14.1 software was used for multivariate statistical analysis of amino acid data. The orthogonal partial least squares (OPLS) approach was used to evaluate the data to screen out the relevant information among the structure and antioxidant properties of the samples.

### Statistical Analysis

All measurements were performed in triplicate, and the results are expressed as mean ± SD. Data were analyzed using JMP software (Trial Version 13.2.1; SAS Institute Inc., Cary, NC, United States) to apply analysis of variance (ANOVA) combined with Student’s *t*-test. GraphPad Prism 8 was adopted to calculate the concentration of black bean protein hydrolysate that exhibited 50% antioxidant activity (IC_50_ value). The graphical representation was analyzed using OriginPro 9.1 (Origin Lab Inc., United States). A *p*-value < 0.05 was considered statistically significant.

## Results and Discussion

### Optimization of Black Bean Protein Hydrolysate

It has been widely accepted that hydrolysis parameters play pivotal roles in the antioxidant capacity of protein hydrolysates (12). To obtain the desirable protein hydrolysates, the optimization of hydrolysis parameters is deemed imperative. As shown in Table 1, the experimental data were collected with 26 combinations of the independent variables, including temperature, pH, enzyme/substrate ratio (E/S), and hydrolysis time. The optimal explanatory model equations were established for DPPH radical scavenging activity, Fe^2+^ chelating activity, and surface hydrophobicity, respectively, using actual values as shown in Eqs. 5–7.


(5) $$\begin{array}{ccc} Y_{1}\left(\%\right)=126.76+4.15x_{1}+10.28x_{2}5.83x_{3}+21.07x_{4} & & \\ +5.95\,x_{1}\,x_{2}+9.31\,x_{1}\,x_{3}+16.19\,x_{1}\,x_{4}+8.10\,x_{2}\,x_{3} & & \\ +13.31\,x_{2}\,x_{4}+11.56\,x_{3}\,x_{4}-28.57\,x_{1}\,2+34.58\,x_{2}\,2 & & \\ -13.83\,x_{3}\,2-1.36\,x_{4}\,2 & & \end{array}$$

(6) Y2(%)=80.75+18.55x1-35.27x3+12.92x4 37.16x1x2-56.04⁢x1⁢x3-20.26⁢x1⁢x4+51.06⁢x2⁢x3-3.95⁢x2⁢x4-2.42⁢x3⁢x4+6.12⁢x1⁢2+39.54⁢x2⁢2-8.96⁢x3⁢2+36.70⁢x4⁢2

(7) Y3=311.36-11.19⁢x1-8.04⁢x2⁢ 18.98⁢x3+4.83⁢x4+5.23⁢x1⁢x2+35.63⁢x1⁢x3+25.21⁢x1⁢x4+14.60⁢x2⁢x3-18.01⁢x2⁢x4-12.80⁢x3⁢x4+54.95⁢x1⁢2+20.30⁢x2⁢2-8.19⁢x3⁢2+54.40⁢x4⁢2

**TABLE 1:** Experimental data of the antioxidant capacity for black bean protein hydrolysate treated with ficin from the central composition design.

Run	Independent variables										
	
	Coded				Actual				Dependent variables		
			
	x_1_	x_2_	x_3_	x_4_	T (°C)	pH	E/S (%)	Time (h)	*Y*_1_ (IC_50_, μ g/mL)	*Y*_2_ (IC_50_, μ g/mL)	*Y*_3_
1	−1	−1	−1	1	50	6.5	1.5	5	111.633.6hi	235.22.29b	528.4913.79a
2	1	−1	−1	1	60	6.5	1.5	5	121.636.07gh	415.126.80a	490.903.28bc
3	−1	1	−1	−1	50	7.5	1.5	3	126.77.46gh	51.250.39*l*m	488.192.45bc
4	1	1	1	1	60	7.5	2.5	5	213.576.58a	52.80.78klm	454.6511.09def
5	−1	−1	−1	−1	50	6.5	1.5	3	150.371.50de	152.091.63ef	518.4910.42ab
6	−1	1	−1	1	50	7.5	1.5	5	124.477.62fgh	129.41.48fgh	472.028.36cd
7	1	1	1	−1	60	7.5	2.5	3	86.923.07jk	81.321.36ijk	452.544.28def
8	1	−1	1	1	60	6.5	2.5	5	126.681.59fg	118.9712.91gh	463.2019.85cde
9	−1.5	0	0	0	47.59	7	2	4	58.310.79n	69.504.25kl	436.9923.60efg
10	−1	1	1	−1	50	7.5	2.5	3	81.80.70klm	188.071.18cd	442.037.21def
11	0	0	0	−1.5	55	7	2	2.52	81.323.97klm	150.934.76ef	435.3417.34efg
12	−1	−1	1	−1	50	6.5	2.5	3	83.752.06klm	80.881.41jk	408.63.63ghi
13	0	0	0	1.5	55	7	2	5.48	168.52.52c	168.811.45de	424.565.20fgh
14	1.5	0	0	0	62.41	7	2	4	71.91.49mn	115.813.11gh	425.3614.56fgh
15	1	1	−1	1	60	7.5	1.5	5	163.172.25cd	158.7316.34def	400.365.39hij
16	0	−1	1	1	50	6.5	2.5	5	89.561.06jk	150.4719.52ef	385.622.05ijk
17	0	0	0	0	55	7	2	4	136.93.67ef	100.046.15hij	314.589.19no
18	0	0	0	0	55	7	2	4	112.53.06ghi	97.048.28hij	311.658.01o
19	1	−1	−1	−1	60	6.5	1.5	3	99.341.52ij	426.0714.52a	367.672.80jkl
20	1	−1	1	1	60	6.5	2.5	3	72.270.80lmn	110.61.68ghi	372.145.90jkl
21	1	1	−1	−1	60	7.5	1.5	3	86.760.59jkl	171.1712.26cde	357.771.68klm
22	0	1.5	0	0	55	7.74	2	4	218.7317.58a	132.2351.98fg	347.216.12*l*mn
23	0	−1.5	0	0	55	6.26	2	4	189.18.25b	2002.17c	362.818.08kl
24	−1	1	1	1	50	7.5	2.5	5	130.633.87ef	177.814.90cde	323.4212.71mno
25	0	0	−1.5	0	55	7	1.26	4	106.63.72i	161.9321.21de	315.619.81no
26	0	0	1.5	0	55	7	2.74	4	88.425.48jk	35.841.36m	269.176.51p

*Y_1_: DPPH scavenging activity; Y_2_: Fe^2+^chelating activity; Y_3_: surface hydrophobicity (H_O_).*

*Different letters indicate the significant difference (P < 0.05) in terms of each dependent variables.*

Where *Y_1_*represents DPPH radical scavenging activity (IC_50,_ μg/mL), *Y*_2_is Fe^2+^ chelating activity (IC_50_, μg/mL), *Y_3_* is surface hydrophobicity, and *x_1_*, *x_2_*, *x_3_*, and *x*_4_represent temperature, pH, E/S, and time, respectively. All models were determined using the ANOVA in Supplementary Tables 1–3. These demonstrate the high significance of the models for DPPH radical scavenging activity, Fe^2+^ chelating activity, and surface hydrophobicity (*P* < 0.001). Furthermore, the coefficients of determination (R^2^) for these three mathematical models were 0.9522, 0.9670, and 0.9242, respectively. These values therefore explain 95.22, 96.70, and 92.42% of behavior variation for DPPH radical scavenging activity, Fe^2+^ chelating activity, and surface hydrophobicity, respectively. The insignificant “lack-of-fit” values further confirmed the validity of the regression modeling procedures.

**FIGURE 1:**
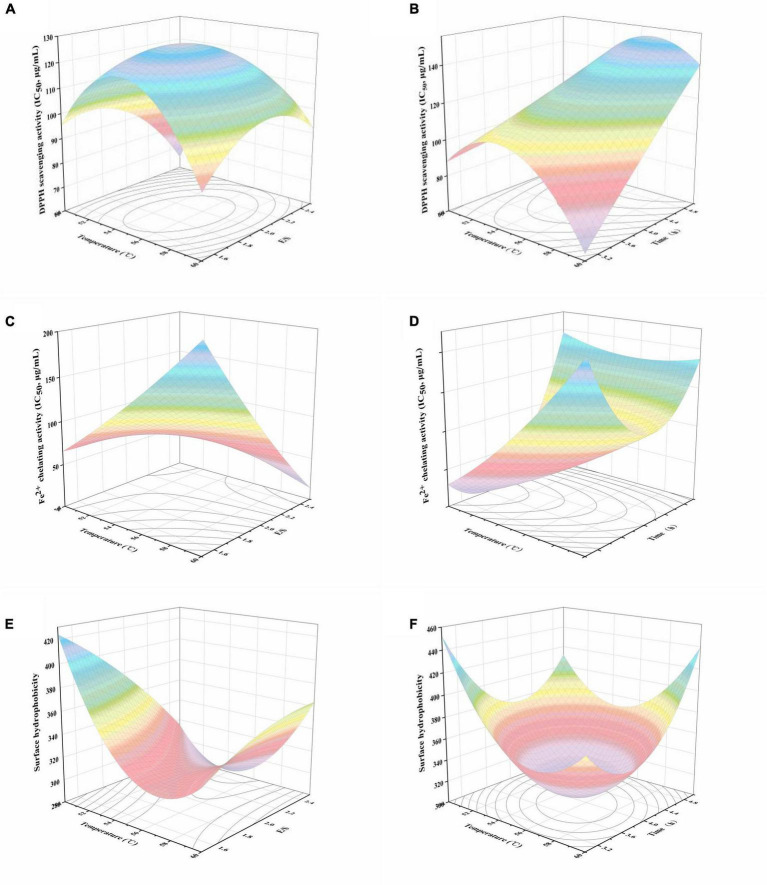
Response surface plots of the effects of temperature and E/S, temperature and time on DPPH radical scavenging activity **(A,B)**, Fe^2+^ chelating activity **(C,D)** and surface hydrophobicity values **(E,F)** of black bean protein hydrolysate.

The response surface plots constructed using the models for DPPH radical scavenging activity, Fe^2+^ chelating activity, and surface hydrophobicity are depicted in Figure 1. DPPH radical scavenging activity and Fe^2+^ chelating activity are presented as IC_50_. With regard to the DPPH radical scavenging activity, quadratic trends were observed for the interaction effects between temperature and E/S or hydrolysis time. As shown in Figure 1A, the increase of both E/S and temperature was conducive to decrease the DPPH radical scavenging activity. The lowest activity occurred at approximately 2% (E/S) and 55 °C, after which the activity increased with higher values of E/S and temperature. Conversely, the prolongation of hydrolysis time led to an appreciable decline in DPPH radical scavenging activity, regardless of the observed temperature fluctuation (Figure 1B). As shown in Figure 1C, the IC_50_ value of Fe^2+^ chelating activity was highest either at the combination of low temperature and high E/S or at high temperature and low E/S. Understandably, the strongest activity was achieved with the combination of both high temperature and E/S. On the contrary, the highest Fe^2+^ chelating activity was observed at the junction of both low temperature and hydrolysis time (Figure 1D).

It is worth noting that peptide surface hydrophobicity has commonly been recognized as an important positive influential factor affecting the antioxidant activity of protein hydrolysates and peptides (13). Surface hydrophobicity increased as a function of E/S at high temperature (Figure 1E). Surface hydrophobicity declined with increasing temperature until approximately 54 °C–57 °C. At this point, surface hydrophobicity increased with further increases in temperature (Figure 1F). Interestingly, hydrolysis time showed a similar tendency to surface hydrophobicity, with the lowest value at a hydrolysis time of about 3.6–4.4 h. This could be due to the exposure or burial of the hydrophobic amino acid residues, which changed with the progression of enzymatic hydrolysis (14).

Taken overall, the optimal conditions appeared to be a temperature of 52°C, pH 7.0, E/S ratio of 2.2, and hydrolysis time of 4.0 h, using JMP software. Further verification tests were conducted with these suggested optimal parameters. Optimal results were determined to be a DPPH radical scavenging activity level of 100.08 ± 2.42 μg/mL, Fe^2+^ chelating activity of 71.49 ± 0.81 μg/mL, and a surface hydrophobicity value of 336.31 ± 6.45. These are very close to the predicted results of DPPH radical scavenging activity (104.71 μg/mL), Fe^2+^ chelating activity (69.42 μg/mL), and surface hydrophobicity values (H_*o*_) (325.50).

### Structural Characteristics of Fractionated Hydrolysates

#### Ultraviolet-Visible Spectra

**FIGURE 2:**
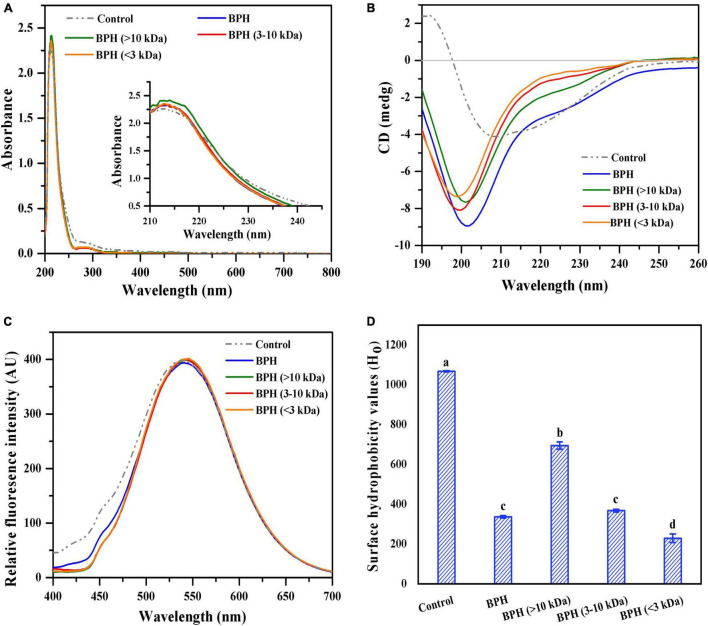
UV-visible spectra of BPH and its membrane fractions **(A)**; CD spectra of BPH and its membrane fractions **(B)**; Fluorescence spectra of BPH and its membrane fractions **(C)**; Surface hydrophobicity values (H_*o*_) of BPH and its membrane fractions **(D)**. Different letters indicate the significant difference (*P* < 0.05).

As described previously, the optimized black bean protein hydrolysate (BPH) exhibited excellent antioxidative capability, which might be associated with structural characteristics like MW (15). BPH was fractionated into different peptide fractions to investigate the relationship between structural properties and antioxidant activity. The ultraviolet-visible spectra of BPH and its peptide fractions were monitored across wavelengths ranging from 200 to 800 nm. As shown in Figure 2A, black bean protein treated with inactivated bromelain (control) displayed a weak absorption at about 280 nm and a strong absorption shoulder at around 210–220 nm. These results corresponded to the findings of Chen et al. (16). When compared to the control group, BPH and its peptide fractions showed a clear decline of absorption peak at 280 nm, signifying protein degradation. Furthermore, elevated absorption bands around 210–220 nm were observed with BPH and its peptide fractions, indicating an increased peptide carboxylic function (16). Interestingly, similar ultraviolet-visible shapes were evident with all hydrolysate samples excluding BPH (>10 kDa), which exhibited the strongest absorption peak at around 210–220 nm. The notable rise of intensity for BPH (>10 kDa) may be attributed to its polypeptide chains containing C = O, −COOH, and CONH2 groups (17).

#### Circular Dichroism Spectroscopy

Circular Dichroism (CD) spectroscopy is widely perceived as an effective technique for investigating the secondary structures of proteins. Thus, we adopted CD spectroscopy to examine the secondary structure changes of protein hydrolysates with varied MWs. As described in Figure 2B, black bean protein treated with inactivated protease (control) showed a strong positive peak at around 192 nm and a prominent negative peak at approximately 200–210 nm, indicating a highly ordered structure with an α-helix. All bromelain-treated hydrolysates presented different CD patterns in comparison to the control, suggesting a loss of the α-helical structure and an increase of β-sheets and random coils. Similar CD results were found in sunflower protein treated with alcalase and whey protein treated with alcalase (18). Intriguingly, the decreased MW resulted in a remarkable blue shift of the negative peak, of which BPH (<3 kDa) showed the lowest ellipticity at the minimum emission wavelength of 199 nm. This could be partially due to shortened peptide chains, helping the resultant peptides form more disordered and flexible structures. These new conformations with high β-types (β-sheet and β-turn) may be favorable for antioxidative capability (19).

#### Fluorescence Spectra

Given the differing secondary structures of hydrolysates with different MW distribution, we reason that BPH and its peptide fractions would also differentiate from each other at the level of the tertiary structure. Thus, to confirm this, we investigated the impact of MW on tertiary structure of the protein hydrolysate through fluorescence emission spectra of ANS across a wavelength range of 400–700 nm. As depicted in Figure 2C, the highest FI of black bean protein treated with inactivated bromelain (control) was recorded at a wavelength of around 540 nm, which corresponded to our previous study (8). Compared with the control, there was a notable red shift in the maximum emission wavelengths for all hydrolysate samples, demonstrating the peptides in those hydrolysates may have undergone conformational changes (20). This red shift could be due to exposure of aromatic amino acid residues (e.g., tryptophan, tyrosine, and phenylalanine) to the solutions (21). Moreover, all protein hydrolysates presented almost overlapping fluorescence emission spectra. Noticeably, the maximum FI of the peptide fractions was superior to that of BPH, implying an increased exposure of hydrophobic amino acids after fractionation.

#### Surface Hydrophobicity

Generally speaking, the relative FI of protein or hydrolysate is closely correlated with surface hydrophobicity (22). Based on this, the surface hydrophobicity values (H_*o*_) of BPH and its peptide fractions were measured and are shown in Figure 2D. All hydrolysate samples presented much lower values of surface hydrophobicity in comparison to control (*P* < 0.05). This is due to bromelain hydrolysis, which leads to buried hydrophobic groups inside the protein cluster as well as more exposed hydrophilic amino acid residues in the solvent (23). Among the three investigated fractions, the surface hydrophobicity significantly increased as a function of MW (*P* < 0.05), with the highest H_*o*_ of 694.46. This presents the probability of a peptide size dependence. Under equivalent conditions, the shortening of peptide chains conferred great flexibility onto the resultant peptides, favoring them to adopt a conformation with outwardly exposed polar amino acid residues (24). Taken together, the conformation and hydrophobic contribution of amino acids played significant roles in the surface hydrophobicity of BPH and its peptide fractions.

### Amino Acid Profiles of Fractionated Hydrolysates

The amino acid profiles of BPH and its peptide fractions were measured and listed in Table 2. Glutamic acid and aspartic acid accounted for approximately 21–25 and 13–15% of the total amino acids in all black bean samples, respectively. This supports previous findings that glutamic acid and aspartic acid are the predominant amino acids of black bean protein isolates (25). Compared to unhydrolyzed black bean protein (control), BPH and its fractions had significantly higher proportions of glutamic acid (*P* < 0.01) but slightly lower contents of aspartic acid. This validates that enzymatic hydrolysis can change amino acid patterns (26). In addition, enzymatic degradation led to increasing proportions of hydrophilic amino acids (>35%), like glutamic acid, tyrosine, glycine, and threonine, imparting an elevated chelation capability to BPH and its peptide fractions (27). Noticeably, the amino acid pattern of BPH (<3 kDa) was significantly distinguishable from BPH (>10 kDa) and BPH (3–10 kDa). More specifically, BPH (<3 kDa) possessed the highest content of hydrophobic amino acids (leucine, valine, phenylalanine, etc.), accounting for more than a quarter of the total amino acids (25.74%). Numerous studies have demonstrated that hydrophobic amino acid residues may be favorable for peptides to readily scavenge lipidic free radicals (28, 29). Taken together, we deduced that smaller MW peptide fractions might improve the ability of protein hydrolysates to scavenge free radicals and chelate metal ions.

**TABLE 2:** Amino acid composition of BPH and its peptide fractions.

Amino acid	BPH (%)	BPH (>10 kDa, %)	BPH (3–10 kDa, %)	BPH (<3 kDa, %)	Control*^a^* (%)
**Hydrophilic amino acids**					
Aspartic acid	14.260.05a	14.720.27ab	14.340.25b	13.270.55c	15.110.51a
Glutamic acid	23.920.29ab	24.590.45a	24.040.40ab	23.300.70b	21.290.83c
Serine	4.860.13a	4.620.18b	4.740.10ab	4.850.12a	4.540.16b
Histidine	2.730.12a	2.570.34a	2.850.13a	2.690.23a	2.680.16a
Glycine	4.380.06ab	4.330.17b	4.420.13ab	4.550.17a	4.360.14ab
Threonine	3.260.02a	3.230.08a	3.270.04a	3.360.13a	3.250.11a
Arginine	7.560.09a	7.230.19b	7.510.10a	7.190.22b	7.060.12b
Tyrosine	4.270.47b	4.170.67b	4.630.29b	4.541.03b	5.850.62a
Cystine	1.211.10b	1.060.79b	1.860.82b	1.941.18b	4.502.80a
Isoleucine	4.000.04bc	4.100.14ab	3.950.13bc	3.870.07c	4.210.20a
Lysine	5.400.78a	4.980.41ab	4.710.32ab	4.100.46bc	3.240.95c
**Hydrophobic amino acid**					
Alanine	3.340.03b	3.150.10c	3.360.04b	3.670.17a	3.420.13b
Valine	4.210.09b	4.160.18b	4.300.07ab	4.460.18a	4.500.20a
Methionine	0.220.19ab	0.310.36ab	0.210.05ab	0.500.16a	0.120.06b
Phenylalanine	4.850.01a	4.730.24a	4.700.28a	4.800.17a	4.940.23a
Leucine	6.190.02b	6.010.11b	6.100.13b	6.500.18a	6.610.30a
Proline	5.341.29a	6.051.48a	5.021.20a	6.401.45a	4.341.71a

*^a^The control group was prepared under the same conditions of the hydrolysate with the addition of inactivated protease. Different letters indicate the significant difference (P < 0.05) in terms of each amino acid.*

### Antioxidant Properties of Fractionated Hydrolysates

#### DPPH Radical Scavenging Activity

We have demonstrated the strong antioxidant properties of the optimized BPH. However, the influence of its structural characteristics (e.g., MW) remains to be unclear. Thus, the impact of MW distribution on antioxidant properties, such as free radicals scavenging activity and metal chelating activity, was investigated.

**FIGURE 3:**
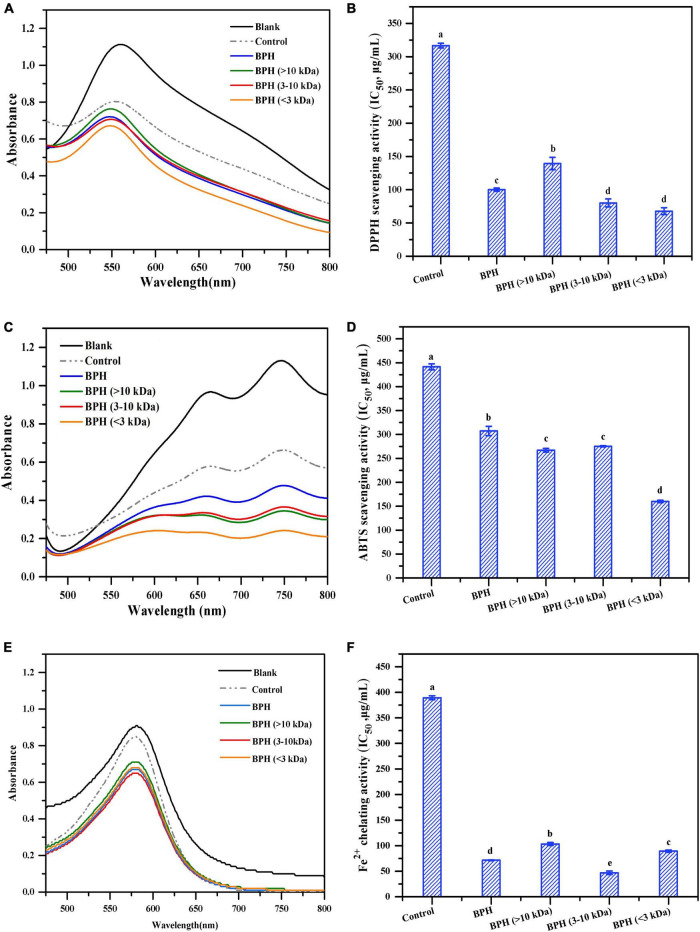
DPPH radical scavenging activity **(A,B)**, ABTS radical scavenging activity **(C,D)** and Fe^2+^ chelating activity **(E,F)** of BPH and its membrane fractions. Different letters indicate the significant difference (*P* < 0.05).

DPPH radical scavenging activity assays have been widely employed to evaluate antioxidative properties of compounds such as hydrogen donors or free radical scavengers (30). As shown in Figure 3A, the spectrum of DPPH radicals was apparently higher than that of unhydrolyzed black bean protein (control group), which showed a much higher absorbance than those of its hydrolysate and peptide fractions. The reduction in spectral absorbance indicated quenching of DPPH radicals induced by BPH and its fractions, supporting the findings of Sun et al. (31). Intriguingly, the quenching effects of DPPH radicals were notably enhanced as the MWs of peptide fractions decreased. A similar variation trend was observed in the IC_50_ values of BPH and its peptide fractions (Figure 3B). Among these three different MW hydrolysates, BPH (<3 kDa) exhibited the highest antioxidant activity with an IC_50_ value of 67.78 μg/mL. Again, this reinforces the concept that smaller peptides seem to possess higher antioxidant potential (32). This is likely attributed to the chain-breaking reaction, delayed initiation, or enhanced termination of low MW hydrolysates, thereby inhibiting free radical-mediated peroxidation of linoleic acid (33). Noticeably, when compared to the unhydrolyzed black bean protein with an IC_50_ value of 316.7 μg/mL, BPH required less than half of that concentration to scavenge 50% of DPPH radicals (*P* < 0.05). Accordingly, optimal hydrolysis was beneficial to elevate the DPPH radical scavenging activity of protein (34).

#### ABTS Radical Scavenging Activity

In addition to the DPPH radical scavenging activity, ABTS radical scavenging activity has been well-documented as another measurement for evaluating the free radical scavenging activity of antioxidant compounds (35). Thus, we investigated the ABTS radical scavenging activity of BPH and its peptide fractions. As shown in Figure 3C, all black bean protein and hydrolysates presented much lower absorbance than that of ABTS radicals, demonstrating their significant antioxidant effects. Interestingly, in comparison with BPH, the three peptide fractions exerted much stronger capacity for scavenging ABTS radicals, emphasizing the considerable relationship between MW distribution and free radical scavenging activity. Among the three BPH fractions, BPH (<3 kDa) showed the strongest ABTS radical scavenging activity, mirroring the DPPH radical scavenging activity results. As shown in Figure 3D, the varying IC_50_ tendencies of ABTS radical scavenging activity were found to be compatible with the spectral changes of all protein and hydrolysates, corresponding to the findings of Wang et al. (36). Upon hydrolysis with bromelain, the significantly enhanced ABTS radical scavenging capabilities were observed in all hydrolysates (*P* < 0.05), suggesting that enzymatic hydrolysis favors the release of peptide fractions with the capacity of scavenging ABTS radicals. The IC_50_ value of BPH (<3 kDa) (160.03 μg/mL) was approximately one-third of the concentration of untreated black bean protein required to scavenge half of the ABTS radicals. This indicates a stronger hydrogen atom transferability and electron transfer capacity of small peptide fractions (37).

#### Metal Chelating Activity

Protein hydrolysates and peptides are widely known to have the potential for chelating transitional metal ions as well as scavenging free radicals (38). In this study, the chelating properties of hydrolysates were evaluated by monitoring the formation of ferrozine and Fe^2+^ ion complexes. As observed in Figure 3E, both black bean protein and hydrolysates corresponded with lower absorbances than that of Fe^2+^ chelating radicals. A similar pattern was also observed with the IC_50_ values of BPH and its peptide fractions (Figure 3F). As expected, unhydrolyzed protein (control) required much higher concentrations to chelate the ferrous ion as compared with protein hydrolysate and its fractions. The exposure of charged groups like aspartic acid and glutamic acid may have contributed to the binding of hydrolysate and its fractions to ferrous ions (39). Interestingly, among all the membrane fractionated samples, the peptide fraction with MW of 3–10 kDa displayed the lowest IC_50_ values (46.85 μg/mL), which was less than one-eighth of that observed in the control group. We speculated that the leading chelating capacity observed for 3–10 kDa peptides might be attributed to their high content of hydrophilic amino acids like histidine, thereby entrapping the transition metals (40).

#### Antioxidant Activity of Black Bean Hydrolysates on Lipid Oxidation

**FIGURE 4:**
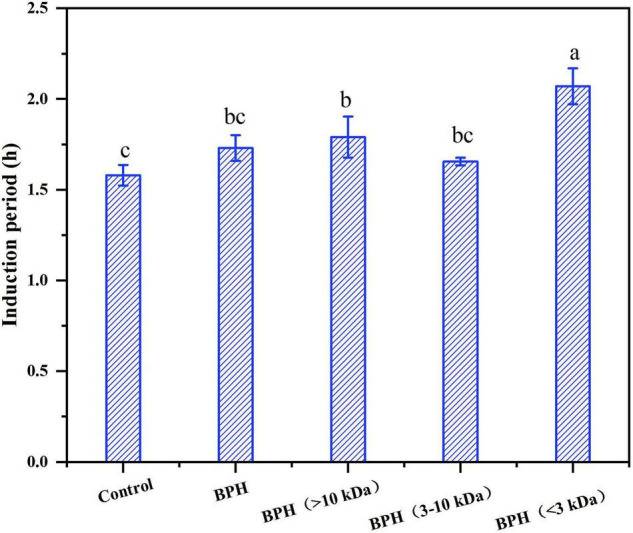
The induction period (IP) of sunflower oil with hydrolysates. Different letters indicate the significant difference (*P* < 0.05).

Since these black bean protein hydrolysates exhibited excellent antioxidant capacity, their antioxidant potential in sunflower oil were then investigated by using rancimat stability assay. As shown in Figure 4, pure sunflower oil (control) showed induction period (IP) of 1.58 ± 0.06 h, the peptide fraction measuring at >10 kDa and <3 kDa had the capacity to prolong the induction period of sunflower oil. Specifically, BPH (3–10 kDa) and BPH (>10 kDa) presented higher IPs than control with 1.66 ± 0.02 h and 1.79 ± 0.11 h, respectively, while BPH showed induction period of 1.73 ± 0.07 h. Similarly, BPH (<3 kDa) slowed down oxidation up to 124.2 ± 5.93 min, performing better potential of retarding the lipid oxidation. This result was consistent with previous results that peptides smaller than 3 kDa exhibited the strongest DPPH and ABTS radical scavenging activity. This may be due to the higher proportions of hydrophobic amino acids compared to the other two fractions.

### Correlation of Molecular Weight, Amino Acids, and Antioxidant Properties of Fractionated Hydrolysates

**FIGURE 5:**
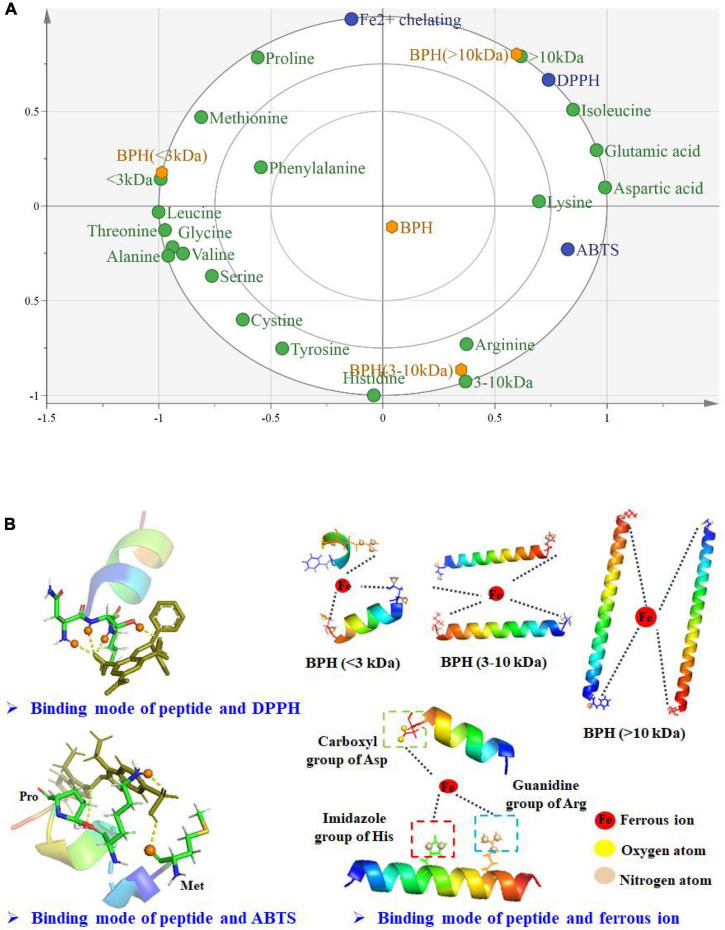
Biplot for BPHs of the correlation of molecular weight, amino acids, and antioxidant properties **(A)**; Simplified antioxidant model of fractionated hydrolysates **(B)**.

To characterize the relationship between structure and antioxidant properties of protein hydrolysate, OPLS analysis was performed using MW and amino acids as the X-matrix, and ABTS, DPPH radical scavenging activity, Fe^2+^ chelating activity (IC_50_, μg/mL) and H_*o*_ as the Y-matrix. As shown in Figure 5A, BPH (<3 kDa) was located on the left side, while BPH (>10 kDa) and BPH (3–10 kDa) were located on the right side, demonstrating the significant differences between small and large MW peptide fractions. Noticeably, the IC_50_ values of ABTS, DPPH radical scavenging activity, and Fe^2+^ chelating activity were significantly positively influenced by lysine, isoleucine, glutamic acid, and aspartic acid in BPH (>10 kDa), but negatively correlated with other amino acids, particularly the hydrophobic amino acids. This could at least partially account for the weaker antioxidant properties of BPH (>10 kDa) among the three investigated membrane fractions. Unexpectedly, BPH (3–10 kDa) was highly positively correlated with histidine and arginine, which could be responsible for the stronger Fe^2+^ chelating activity. Interestingly, small peptides with MW < 3 kDa exhibited a negative correlation with IC_50_ values of ABTS and DPPH radical scavenging activity, further highlighting their excellent antioxidant characteristics. This is likely because of the hydrophobic aliphatic amino acids, such as methionine, leucine, valine, and alanine, which may promote the antioxidant activities of protein hydrolysates or fractions (28).

### Simplified Antioxidant Models of Fractionated Hydrolysates

As mentioned above, black BPHs and their peptide fractions exhibit a remarkable capacity to scavenge free radicals and chelate metal ions. To vividly interpret the antioxidant patterns, simplified models of black bean protein hydrolysate/fractions on free radicals and metal ion were constructed and shown in Figure 5B. The amino acid residues present in protein hydrolysate/fractions could effectively form hydrogen bonds with DPPH or ABTS molecules. In other words, these free radicals could accept hydrogen atoms donated from BPH and peptide fractions in order to become stable molecules. Under equivalent conditions, BPH (<3 kDa) probably has more opportunity to transfer hydrogens to free radicals when compared to larger peptide fractions. In addition, different MWs conferred various Fe^2+^ chelating activities on the peptide fractions, likely associated with their peptide sizes and amino acid residues. Specifically, the active binding sites in protein hydrolysate/fractions might surround the ferrous ions by forming Fe-N and Fe-O bonds (41). Ferrous ions might bind with fractionated hydrolysates primarily *via* interactions with nitrogen atoms in the imidazole group of histidine, guanidine, and arginine as well as the carboxyl oxygen of aspartic acid. Accordingly, their high contents may contribute to the strong capability of BPH (3–10 kDa) to chelate ferrous ions, thereby terminating the oxidative reaction mediated by transition metal ions.

## Conclusion

This study implemented bromelain hydrolysis to facilitate the antioxidant activities of hydrolysate and its peptide fractions from black bean protein. Under the established optimal hydrolysis parameters (temperature 52°C, pH 7, E/S ratio 2.2, hydrolysis time 4 h), the optimized black BPH showed a surface hydrophobicity value of 336.31 ± 6.45 and required a concentration of 100.08 ± 2.42 and 71.49 ± 0.81 μg/mL to scavenge 50% of DPPH radical and to chelate 50% of ferrous ion, respectively. Spectral analysis revealed that the MW distributions exerted key roles in the structural changes of BPH and the resulting peptide fractions. Moreover, the antioxidant properties of the investigated peptide fractions were deemed superior to the unhydrolyzed protein and even BPH. Intriguingly, the peptide fraction of 3–10 kDa showed the highest Fe^2+^ chelating activity, while the smaller peptides <3 kDa had the optimal capability to scavenge DPPH and ABTS radicals. Moreover, the peptide fraction with MW < 3 kDa could predominantly prolong the storage period of sunflower oil about 78 days. This could be due to either the high contents of histidine and arginine in the 3–10 kDa fraction or the hydrophobic aliphatic amino acids in smaller peptides. Overall, this evidence strongly suggests that black bean protein hydrolysate/fractions with low MW are deemed advantageous for developing food products and preventing food oxidation.

## Data Availability Statement

The original contributions presented in the study are included in the article/Supplementary Material, further inquiries can be directed to the corresponding author.

## Author Contributions

YC: conceptualization, methodology, data curation, and writing – original draft preparation. ZZ: writing – reviewing and editing. ZA and YZ: investigation. YL: supervision and validation. All authors contributed to the article and approved the submitted version.

## Conflict of Interest

YL was employed by the company Future Food (Nanjing Fuzhe) Research Institute Co., Ltd. The remaining authors declare that the research was conducted in the absence of any commercial or financial relationships that could be construed as a potential conflict of interest.

## Publisher’s Note

All claims expressed in this article are solely those of the authors and do not necessarily represent those of their affiliated organizations, or those of the publisher, the editors and the reviewers. Any product that may be evaluated in this article, or claim that may be made by its manufacturer, is not guaranteed or endorsed by the publisher.

## References

[1] Sebastiani G Barbero AH Borrás-Novell C Casanova MA Aldecoa-Bilbao V Andreu-Fernández V The effects of vegetarian and vegan diet during pregnancy on the health of mothers and offspring. *Nutrients.* (2019) 11:557. 10.3390/nu11030557 30845641 PMC6470702

[2] Jiang L Wang J Li Y Wang Z Liang J Wang R Effects of ultrasound on the structure and physical properties of black bean protein isolates. *Food Res Int.* (2014) 62:595–601. 10.1016/j.foodres.2014.04.022

[3] Marson GV Machado MC Janser S Hubinger M. Sequential hydrolysis of spent brewer’s yeast improved its physico-chemical characteristics and antioxidant properties: a strategy to transform waste into added-value biomolecules. *Process Biochem.* (2019) 84:91–102. 10.1016/j.procbio.2019.06.018

[4] Ng KL Ayob MK Said M Osman MA Ismail A. Optimization of enzymatic hydrolysis of palm kernel cake protein (PKCP) for producing hydrolysates with antiradical capacity. *Industrial Crops Prod.* (2013) 43:725–31. 10.1016/j.indcrop.2012.08.017

[5] Wen C Zhang J Zhang H Duan Y Ma H. Plant protein-derived antioxidant peptides: isolation, identification, mechanism of action and application in food systems: a review. *Trends Food Sci Technol.* (2020) 105:308–22. 10.1016/j.tifs.2020.09.019

[6] Zhe L Zhai A. Preparation and preliminary separation of antioxidant peptide of rice bran protein. *J Hlongjiang Bayi Agric Univ.* (2012) 24:56–59.

[7] Zou Z Wang M Wang Z Aluko RE He R. Antihypertensive and antioxidant activities of enzymatic wheat bran protein hydrolysates. *J Food Biochem.* (2019) 44:e13090. 10.1111/jfbc.13090 31663146

[8] Zheng Z Li J Li J Sun H Liu Y. Physicochemical and antioxidative characteristics of black bean protein hydrolysates obtained from different enzymes. *Food Hydrocolloids.* (2019) 97:105222.1–9.

[9] Ngoh YY Gan CY. Enzyme-assisted extraction and identification of antioxidative and α-amylase inhibitory peptides from Pinto beans (Phaseolus vulgaris cv. Pinto). *Food Chem.* (2016) 190:331–7. 10.1016/j.foodchem.2015.05.120 26212978

[10] Huang CY Tsai YH Hong YH Hsieh SL Huang RH. Characterization and antioxidant and angiotensin I-Converting Enzyme (ACE)-Inhibitory activities of gelatin hydrolysates prepared from extrusion-pretreated milkfish (Chanos chanos) scale. *Marine Drugs.* (2018) 16:346. 10.3390/md16100346 30248998 PMC6213483

[11] Abdualrahman MAY Zhou C Zhang Y ElGasim A. Effects of ultrasound pretreatment on enzymolysis of sodium caseinate protein: kinetic study, angiotensin-converting enzyme inhibitory activity, and the structural characteristics of the hydrolysates. *J Food Processing Preserv.* (2017) 41:e13276. 10.1111/jfpp.13276

[12] Galante M Flaviis RD Boeris V Spelzini D. Effects of the enzymatic hydrolysis treatment on functional and antioxidant properties of quinoa protein acid-induced gels. *LWT- Food Sci Technol.* (2019) 118:108845. 10.1016/j.lwt.2019.108845

[13] Xie N Wang B Jiang L Liu C Li B. Hydrophobicity exerts different effects on bioavailability and stability of antioxidant peptide fractions from casein during simulated gastrointestinal digestion and Caco-2 cell absorption. *Food Res. Int.* (2015) 76:518–26. 10.1016/j.foodres.2015.06.025 28455033

[14] Ibrahim E Ghani MA. The effect of enzymatic hydrolysis on the antioxidant activities and amino acid profiles of defatted chia (Salvia hispanica L.) flour. *Food Res.* (2020) 4:38–50. 10.26656/fr.2017.4(s4).003

[15] Mhd SN Farah B Howell NK. Purification and characterization of antioxidative peptides derived from chicken skin gelatin hydrolysate. *Food Hydrocolloids.* (2018) 85:311–20. 10.1016/j.foodhyd.2018.06.048

[16] Chen ZH Liu B Zhao LN. Fabrication and characterization of *Grifola frondosa* protein hydrolysate-selenium chelate. *Food Sci Technol Res.* (2020) 26:101–10. 10.3136/fstr.26.101

[17] Abdollahi M Rezaei M Jafarpour A Undeland I. Sequential extraction of gel-forming proteins, collagen and collagen hydrolysate from gutted silver carp (Hypophthalmichthys molitrix), a biorefinery approach. *Food Chem.* (2018) 242:568–78. 10.1016/j.foodchem.2017.09.045 29037731

[18] Wu Q Zhang X Jia J Kuang C Yang H. Effect of ultrasonic pretreatment on whey protein hydrolysis by alcalase: thermodynamic parameters, physicochemical properties and bioactivities. *Process Biochem.* (2018) 67:46–54. 10.1016/j.procbio.2018.02.007

[19] Yuan HN Lv JM Gong JY Xiao GN Zhu RY Li L Secondary structures and their effects on antioxidant capacity of antioxidant peptides in yogurt. *Int J Food Properties.* (2018) 21:2167–80. 10.1080/10942912.2018.1501700

[20] Jian R Song CI Zhang HI Kopparapu NU Zheng XU. Effect of hydrolysis degree on structural and interfacial properties of sunflower protein isolates. *J Food Process Preserv.* (2016) 41:e13092. 10.1111/jfpp.13092

[21] Zhang X Wang L Chen Z Li Y Luo X Li Y. Effect of electron beam irradiation on the structural characteristics and functional properties of rice proteins. *RSC Adv.* (2019) 9:13550–60. 10.1039/c8ra10559f 35519547 PMC9063936

[22] Evangelho JA Vanier NL Pinto VZ Berrios JJ Dias AR Zavareze ER. Black bean (Phaseolus vulgaris L.) protein hydrolysates: physicochemical and functional properties. *Food Chem.* (2017) 214:460–7. 10.1016/j.foodchem.2016.07.046 27507499

[23] Selamassakul O Laohakunjit N Kerdchoechuen O Ratanakhanokchai K. A novel multi-biofunctional protein from brown rice hydrolysed by endo/endo-exoproteases. *Food Funct.* (2016) 7:2635–44. 10.1039/c5fo01344e 27186602

[24] Liu Q Kong B Xiong YL Xia X. Antioxidant activity and functional properties of porcine plasma protein hydrolysate as influenced by the degree of hydrolysis. *Food Chem.* (2010) 118:403–10. 10.1016/j.foodchem.2009.05.013

[25] Chen M Ji H Zhang Z Zeng X Liu S. A novel calcium-chelating peptide purified from Auxis thazard protien hydrolysate and its binding properties with calcium. *J Funct Foods.* (2019) 60:103447. 10.1016/j.jff.2019.103447

[26] Farvin KHS Andersen LL Otte J Nielsen HH Jessen F Jacobsen C. Antioxidant activity of cod (Gadus morhua) protein hydrolysates: fractionation and characterisation of peptide fractions. *Food Chem.* (2016) 204:409–19. 10.1016/j.foodchem.2016.02.145 26988519

[27] O’Loughlin I Kelly PM Murray BA Fitzgerald RJ Brodkorb A. Molecular characterization of whey protein hydrolysate fractions with ferrous chelating and enhanced iron solubility capabilities. *J Agric Food Chem.* (2015) 63:2708–14. 10.1021/jf505817a 25716093

[28] Paiva L Lima E Neto AI Baptista J. Angiotensin I-Converting Enzyme (ACE) inhibitory activity, antioxidant properties, phenolic content and amino acid profiles of Fucus spiralis L. *Protein Hydrolysate Fract. Mar Drugs.* (2017) 15:311. 10.3390/md15100311 29027934 PMC5666419

[29] Ao J Li B. Amino acid composition and antioxidant activities of hydrolysates and peptide fractions from porcine collagen. *Food Sci Technol Int.* (2012) 18:425–34. 10.1177/1082013211428219 23064526

[30] Idowu AT Benjakul S Sinthusamran S Sookchoo P Kishimura H. Protein hydrolysate from salmon frames: production, characteristics and antioxidative activity. *J Food Biochem.* (2019) 43:e12734. 10.1111/jfbc.12734 31353651

[31] Sun X Jia P Zhe T Bu T Liu Y Wang Q Construction and multifunctionalization of chitosan-based three-phase nano-delivery system. *Food Hydrocolloids.* (2019) 96:402–11. 10.1016/j.foodhyd.2019.05.040

[32] Saallah S Ishak NH Sarbon NM. Effect of different molecular weight on the antioxidant activity and physicochemical properties of golden apple snail (Ampullariidae) protein hydrolysates. *Food Res.* (2020) 4:1363–70. 10.26656/fr.2017.4(4).348

[33] Foh MBK Qixing J Amadou I Xia WS. Influence of ultrafiltration on antioxidant activity of Tilapia (Oreochromis niloticus) protein hydrolysate. *Adv J Food Sci Technol.* (2010) 2:227–35.

[34] Ahn CB Kim JG Je JY. Purification and antioxidant properties of octapeptide from salmon byproduct protein hydrolysate by gastrointestinal digestion. *Food Chem.* (2014) 147:78–83. 10.1016/j.foodchem.2013.09.136 24206688

[35] Carrasco-Castilla J Hernandez-Alvarez AJ Jimenez-Martinez C Jacinto-Hernandez C Alaiz M Giron-Calle J Antioxidant and metal chelating activities of peptide fractions from phaseolin and bean protein hydrolysates. *Food Chem.* (2012) 135:1789–95. 10.1016/j.foodchem.2012.06.016 22953924

[36] Wang L Ma M Yu Z Du SK. Preparation and identification of antioxidant peptides from cottonseed proteins. *Food Chem.* (2021) 352:129399. 10.1016/j.foodchem.2021.129399 33662918

[37] He S Zhang Y Sun H Du M Qiu J Tang M Antioxidative peptides from proteolytic hydrolysates of false abalone (Volutharpa ampullacea perryi): characterization, identification, and molecular docking. *Mar Drugs.* (2019) 17:116. 10.3390/md17020116 30781881 PMC6409897

[38] Su S Wan Y Guo S Chao Z Zhang T Ming L. Effect of peptide–phenolic interaction on the antioxidant capacity of walnut protein hydrolysates. *Int J Food Sci Technol.* (2018) 53:508–15. 10.1111/ijfs.13610

[39] Phongthai S D’Amico S Schoenlechner R Homthawornchoo W Rawdkuen S. Fractionation and antioxidant properties of rice bran protein hydrolysates stimulated by in vitro gastrointestinal digestion. *Food Chem.* (2018) 240:156. 10.1016/j.foodchem.2017.07.080 28946256

[40] Zaky AA Chen Z Liu Y Li S Jia Y. Preparation and assessment of bioactive extracts having antioxidant activity from rice bran protein hydrolysates. *J Food Measurement Characterization.* (2019) 13:2542–8. 10.1007/s11694-019-00174-9

[41] Sun N Cui P Jin Z Wu H Wang Y Lin S. Contributions of molecular size, charge distribution, and specific amino acids to the iron-binding capacity of sea cucumber (*Stichopus japonicus*) ovum hydrolysates. *Food Chem.* (2017) 230:627–36. 10.1016/j.foodchem.2017.03.077 28407960

