# Supplementary material for: Exploring the Antioxidant and Structural Properties of Black Bean Protein Hydrolysate and Its Peptide Fractions
Source: Front Nutr. 2022 Jun 6;9:884537. doi: 10.3389/fnut.2022.884537 (PMC9207475; doi:10.3389/fnut.2022.884537)
Supplement: Supplementary file 1 [file Data_Sheet_1.docx]

**Table S1** The model coefficients estimated by multiple linear regression were used to treat the DPPH radical scavenging activity of black soybean protein hydrolysate with bromelain.

| Source | Sum of Squares | DF^a^ | Mean Square | *F* Ratio | Prob > *F* | Significant |
| --- | --- | --- | --- | --- | --- | --- |
| Model | 1372.005 | 14 | 9798.85 | 89.65 | < .0001 | *** |
| Linear effects |  |  |  |  |  |  |
| $x_{1}$ | 1052.14 | 1 | 1052.14 | 9.63 | 0.0029 |  |
| $x_{2}$ | 6468.25 | 1 | 6468.25 | 59.18 | < .0001 | *** |
| $x_{3}$ | 2077.24 | 1 | 2077.24 | 19.01 | < .0001 | *** |
| $x_{4}$ | 27155.87 | 1 | 27155.87 | 248.46 | < .0001 | *** |
| Interaction effects |  |  |  |  |  |  |
| $x_{1}$·$x_{2}$ | 1699.44 | 1 | 1699.44 | 15.55 | 0.0002 |  |
| $x_{1}$·$x_{3}$ | 4159.52 | 1 | 4159.52 | 38.06 | < .0001 | *** |
| $x_{1}$·$x_{4}$ | 12585.78 | 1 | 12585.78 | 115.15 | < .0001 | *** |
| $x_{2}$·$x_{3}$ | 3145.88 | 1 | 3145.88 | 28.78 | < .0001 | *** |
| $x_{2}$·$x_{4}$ | 8497.37 | 1 | 8497.37 | 77.75 | < .0001 | *** |
| $x_{3}$·$x_{4}$ | 6415.11 | 1 | 6415.11 | 58.70 | < .0001 | *** |
| Squared effects |  |  |  |  |  |  |
| $x_{1}^{2}$ | 23664.25 | 1 | 23664.25 | 216.52 | < .0001 | *** |
| $x_{2}^{2}$ | 34666.69 | 1 | 34666.69 | 317.18 | < .0001 | *** |
| $x_{3}^{2}$ | 5542.51 | 1 | 5542.51 | 50.71 | < .0001 | *** |
| $x_{4}^{2}$ | 53.88 | 1 | 53.88 | 0.49 | 0.4852 |  |
| Lack of fit | 1847.79 | 10 | 184.78 | 1.94 | 0.0592 |  |
| Pure error | 5037.81 | 53 | 95.05 |  |  |  |
| Total error | 1441.005 | 77 |  |  |  |  |

^a^ DF, degrees of freedom

**Table S2** The model coefficients estimated by multiple linear regression were used to treat the Fe^2+^ chelating effects of black soybean protein hydrolysate with bromelain.

| Source | Sum of Squares | DF^a^ | Mean Square | *F* Ratio | Prob > *F* | Significant |
| --- | --- | --- | --- | --- | --- | --- |
| Model | 6785.005 | 14 | 48467.10 | 131.77 | < 0.0001 | *** |
| Linear effects |  |  |  |  |  |  |
| $x_{1}$ | 21044.02 | 1 | 21044.02 | 57.21 | < 0.0001 | *** |
| $x_{2}$ | 76100.93 | 1 | 76100.93 | 206.90 | < 0.0001 | *** |
| $x_{3}$ | 1205.005 | 1 | 1205.005 | 327.57 | < 0.0001 | *** |
| $x_{4}$ | 10213.78 | 1 | 10213.78 | 27.77 | < 0.0001 | *** |
| Interaction effects |  |  |  |  |  |  |
| $x_{1}$·$x_{2}$ | 66288.98 | 1 | 66288.98 | 180.22 | < 0.0001 | *** |
|  |  |  |  |  |  |  |
| $x_{1}$·$x_{3}$ | 1507.005 | 1 | 1507.005 | 409.84 | < 0.0001 | *** |
|  |  |  |  |  |  |  |
| $x_{1}$·$x_{4}$ | 19698.39 | 1 | 19698.39 | 53.56 | < 0.0001 | *** |
|  |  |  |  |  |  |  |
| $x_{2}$·$x_{3}$ | 1251.005 | 1 | 1251.005 | 340.25 | < 0.0001 | *** |
|  |  |  |  |  |  |  |
| $x_{2}$·$x_{4}$ | 748.13 | 1 | 748.13 | 2.03 | 0.1588 |  |
|  |  |  |  |  |  |  |
| $x_{3}$·$x_{4}$ | 280.62 | 1 | 280.62 | 0.76 | 0.3857 |  |
|  |  |  |  |  |  |  |
| Squared effects |  |  |  |  |  |  |
| $x_{1}^{2}$ | 1085.73 | 1 | 1085.73 | 2.95 | 0.0907 |  |
| $x_{2}^{2}$ | 45330.81 | 1 | 45330.81 | 123.24 | < 0.0001 | *** |
| $x_{3}^{2}$ | 2325.85 | 1 | 2325.85 | 6.32 | 0.0145 |  |
| $x_{4}^{2}$ | 39046.17 | 1 | 39046.17 | 106.16 | < 0.0001 | *** |
| Lack of fit | 4714.93 | 10 | 471.49 | 1.35 | 0.2274 |  |
| Pure error | 18457.26 | 53 | 348.25 |  |  |  |
| Total error | 7017.005 | 77 |  |  |  |  |

^a^ DF, degrees of freedom

**Table S3** The model coefficients estimated by multiple linear regression were used to treat the surface hydrophobicity values of black soybean protein hydrolysate with bromelain.

| Source | Sum of Squares | DF^a^ | Mean Square | *F* Ratio | Prob > *F* | Significant |
| --- | --- | --- | --- | --- | --- | --- |
| Model | 3487.005 | 14 | 24907.23 | 54.86 | < 0.0001 | *** |
| Linear effects |  |  |  |  |  |  |
| $x_{1}$ | 7659.88 | 1 | 7659.88 | 16.87 | 0.0001 |  |
| $x_{2}$ | 3952.93 | 1 | 3952.93 | 8.71 | 0.0044 |  |
| $x_{3}$ | 22051.83 | 1 | 22051.83 | 48.57 | < 0.0001 | *** |
| $x_{4}$ | 1429.23 | 1 | 1429.23 | 3.15 | 0.0808 |  |
| Interaction effects |  |  |  |  |  |  |
| $x_{1}$·$x_{2}$ | 1310.81 | 1 | 1310.81 | 2.89 | 0.0942 |  |
|  |  |  |  |  |  |  |
| $x_{1}$·$x_{3}$ | 60924.17 | 1 | 60924.17 | 134.20 | < .0001 | *** |
|  |  |  |  |  |  |  |
| $x_{1}$·$x_{4}$ | 30513.98 | 1 | 30513.98 | 67.21 | < .0001 | *** |
|  |  |  |  |  |  |  |
| $x_{2}$·$x_{3}$ | 10234.48 | 1 | 10234.48 | 22.54 | < .0001 | *** |
|  |  |  |  |  |  |  |
| $x_{2}$·$x_{4}$ | 15561.51 | 1 | 15561.51 | 34.28 | < .0001 | *** |
|  |  |  |  |  |  |  |
| $x_{3}$·$x_{4}$ | 7858.79 | 1 | 7858.79 | 17.31 | < .0001 | *** |
|  |  |  |  |  |  |  |
| Squared effects |  |  |  |  |  |  |
| $x_{1}^{2}$ | 87541.14 | 1 | 87541.14 | 192.83 | < 0.0001 | *** |
| $x_{2}^{2}$ | 11947.38 | 1 | 11947.38 | 26.32 | < 0.0001 | *** |
| $x_{3}^{2}$ | 1942.95 | 1 | 1942.95 | 4.28 | 0.0427 |  |
| $x_{4}^{2}$ | 85772.14 | 1 | 85772.14 | 188.93 | < 0.0001 | *** |
| Lack of fit | 7578.94 | 10 | 757.89 | 1.91 | 0.0641 |  |
| Pure error | 21021.72 | 53 | 396.64 |  |  |  |
| Total error | 3773.005 | 77 |  |  |  |  |

^a^ DF, degrees of freedom
